# Lewis Acid-Induced Dinitrogen Cleavage in an Anionic Side-on End-on Bound Dinitrogen Diniobium Hydride Complex

**DOI:** 10.3390/molecules27175553

**Published:** 2022-08-29

**Authors:** Naofumi Suzuki, Yutaka Ishida, Hiroyuki Kawaguchi

**Affiliations:** Department of Chemistry, Tokyo Institute of Technology, Ookayama, Meguro-ku, Tokyo 152-8551, Japan

**Keywords:** dinitrogen complex, niobium, coordination chemistry, hydride complex

## Abstract

The side-on end-on dinitrogen hydride complex [{Na(dme)}_2_{(O_3_)Nb}_2_(μ-η^1^:η^2^-N_2_)(μ-H)_2_] (**3-Na**, [O_3_]^3−^ = [(3,5-^t^Bu_2_-2-O-C_6_H_2_)_3_CH]^3−^) was observed to undergo facile elimination of H_2_ and cleavage of the N–N bond in the presence of 9-borabicyclo[3.3.1]nonane (9-BBN), AlMe_3_, and ZnMe_2_. Treatment of **3-Na** with 9-BBN and ZnMe_2_ afforded the nitride complex [{K(dme)_2_}_2_{(O_3_)Nb}_2_(μ-N)_2_] (**2-Na**). The reaction of **3-Na** with AlMe_3_ afforded [{Na(dme)}_2_{(O_3_)AlMe}_2_(NbMe_2_)_2_(μ-N)_2_] (**5**). The nitride complex **2-Na** was treated with 9-BBN and AlMe_3_ to form [{Na(dme)}_2_{(O_3_)Nb}(μ-NH)(μ-NBC_8_H_14_){Nb(O_3_C)}] (**4**) and **5**, respectively. Complex **2-Na**, **4**, and **5** were structurally characterized.

## 1. Introduction

Activation of dinitrogen under mild conditions has been of long-standing interest in chemistry owing to its industrial and biological significance. In this context, since the discovery of the first dinitrogen complex [Ru(N_2_)(NH_3_)_5_]^2+^ in 1965 [1], a number of dinitrogen complexes have been synthesized and characterized [2,3,4]. A variety of coordination modes have been found in dinitrogen complexes to date, and dinitrogen complexes show diverse reactivity patterns [5,6,7,8,9,10]. With dinuclear complexes, the side-on end-on coordination mode produces substantial polarization of the coordinated dinitrogen [10,11,12,13], and seminal works from Fryzuk have demonstrated that complexes of this type display a rich functionalization chemistry [14]. However, only a limited number of side-on end-on dinitrogen complexes are known [11,15,16,17]. Therefore, this coordination mode becomes one of synthetic targets in dinitrogen chemistry.

The reactions of Lewis acids with dinitrogen complexes provide suitable reference points to evaluate the reactivity of coordinated N_2_ toward electrophiles. The reactions of mononuclear dinitrogen complexes with group 13 Lewis acids are known and frequently proceed at the β-N atom to form stable adducts [18,19,20,21,22]. Addition of group 13 Lewis acids to side-on end-on dinitrogen hydride complexes has also been reported to occur at the β-N atom. For example, Fryzuk described the reaction of [{(NPN)Ta}_2_(μ-η^1^:η^2^-N_2_)(μ-H)_2_] ([NPN] = [PhP(CH_2_SiMe_2_NPh)_2_]^2−^) with EX_3_ (B(C_6_F_5_)_3_, AlMe_3_, and GaMe_3_) to give the corresponding adducts [{(NPN)Ta}_2_(μ-η^1^:η^2^-N_2_EX_3_)(μ-H)_2_] [23]. The analogous titanium complex [{(PNP)Ti}_2_(μ-η^1^:η^2^-N_2_)(μ-H)_2_] ([PNP] = 4,5-bis(diisopropylphosphino)-2,7,9,9-tetramethyl-9H-acridin-10-ide) was also reported to initially form the Lewis acid–base adducts with B(C_6_F_5_)_3_ and AlMe_3_ [16].

We have previously reported the synthesis of a series of anionic dinuclear Nb(IV) hydride complexes containing alkali metal ions [{M(dme)}_2_{(O_3_)Nb}_2_(μ-H)_4_] (**1-M**, M = Li, Na, K, [O_3_]^3−^ = [(3,5-^t^Bu_2_-2-O-C_6_H_2_)_3_CH]^3−^) and the counterion dependence of their reactivity toward N_2_ [24]. The potassium derivative **1-K** readily reacted with N_2_ via the reductive elimination of two equivalents of H_2_ to yield the nitride bridged complex [{K(thf)_2_}_2_{(O_3_)Nb}_2_(μ-N)_2_] (**2-K**) [25]. For the lithium and sodium derivatives, the reactions with N_2_ did not result in the cleavage of the N_2_ triple bond but led to the elimination of only one equivalent of H_2_ and the formation of the corresponding side-on end-on dinitrogen hydride complexes. The lithium salt of the side-on end-on complex was unstable and converted to the end-on bridging dinitrogen complex with concomitant loss of the birding hydride ligands, whereas the sodium salt [{Na(dme)}_2_{(O_3_)Nb}_2_(μ-η^1^:η^2^-N_2_)(μ-H)_2_] (**3-Na**) was stable enough to use as a starting material for further reactivity studies.

The complex **3-Na** is the first example of an anionic side-on end-on dinitrogen complex, and the anionic charge is expected to enhance the nucleophilicity of the β-N atom. We preliminary demonstrated that the reaction of **3-Na** with Me_3_SiCl occurred at the β-N atom of the coordinated N_2_ unit [25]. Therefore, we were interested in the reactions with other Lewis acids to investigate the nucleophilicity of the N_2_ unit in the anionic complex **3-Na**. Here we report the reactions of the side-on end-on dinitrogen hydride complex **3-Na** with ZnMe_2_, AlMe_3_, and 9-borabicyclo[3.3.1]nonane (9-BBN).

## 2. Results

### 2.1. Reaction with 9-BBN

**Scheme 1:**
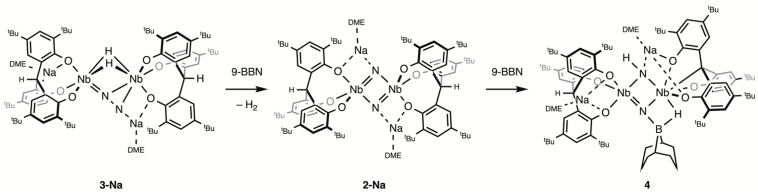
Reaction of **3-Na** with 9-BBN.

Addition of 9-BBN to a green solution of **3-Na** in toluene at room temperature gradually gave a brown solution with concomitant liberation of H_2_, from which [{Na(dme)}_2_{(O_3_)Nb}_2_(μ-N)_2_] (**2-Na**) was isolated as a brown powder in a 67% yield upon workup (Scheme 1). Monitoring the reaction by ^1^H NMR spectroscopy in C_6_D_6_ indicated that 9-BBN was left unreacted. To probe the origin of the eliminated H_2_, the deuterated complex [{Na(dme)}_2_{(O_3_)Nb}_2_(μ-η^1^:η^2^-N_2_)(μ-D)_2_] (**3-Na-D_2_**) was allowed to react with 9-BBN, which resulted in the formation of **2-Na** and D_2_. The complex **3-Na** is stable in toluene in the absence of 9-BBN. These findings indicate that 9-BBN promotes the reductive elimination of H_2_, providing two electrons for N–N bond cleavage to form the nitride complex **2-Na**.

**Figure 1:**
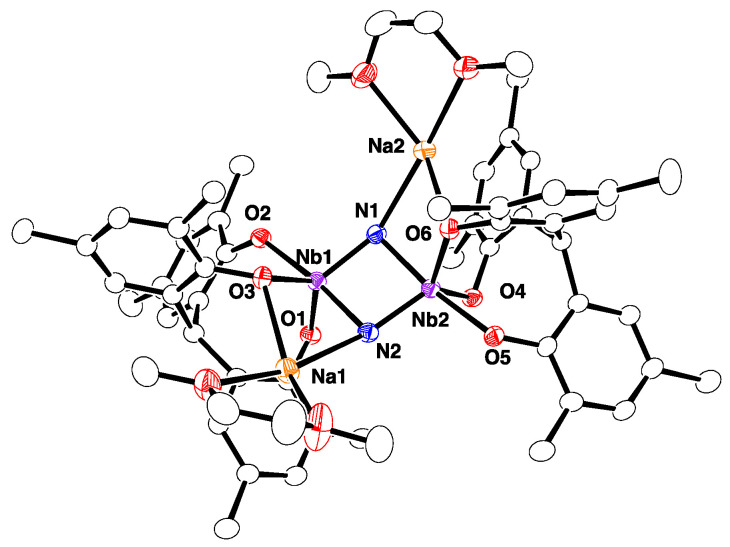
Molecular structure of **2-Na** with thermal ellipsoids set at 30% probability level. All hydrogen atoms and methyl groups of *tert*-butyl groups are omitted for clarity.

The molecular structure of **2-Na**, which was determined by single-crystal X-ray diffraction (Figure 1), revealed the dimer to be bridged by two nitride ligands. The structure of **2-Na** closely resembles that of the potassium salt **2-K** [25]. As with the potassium salt **2-K**, the complex occurs as an ion pair, wherein each DME-solvated sodium cation is coordinated by a nitride group and a phenoxide group. The planar Nb_2_N_2_ core of **2-Na** is asymmetric, such that the Nb–N bond lengths are different (Nb1-N1 = 1.842(2) Å, Nb2–N2 = 1.838(2) Å, and Nb1-N2 = 2.025(2) Å, Nb2–N1 = 2027(2) Å). The average Nb–N bond length of 1.933(2) Å is similar to that of [{Na(dme)}_2_{[*p*-^t^Bu-calix[4]-(O)_4_]Nb}_2_(μ-N)_2_] (average 1.910(8) Å) [26].

Reactions of mono- and dinuclear dinitrogen complexes with hydroboranes were reported to result in B–N bond formation due to the considerable strength of a B–N bond [27,28,29,30]. In the reaction with **2-Na**, 9-BBN was observed to induce H_2_ elimination and N–N bond cleavage rather than undergo N-borylation. This is in contrast with what Fryzuk reported for the reaction of [{(NPN)Ta}_2_(μ-η^1^:η^2^-N_2_)(μ-H)_2_] with 9-BBN, which initially formed [{(NPN)TaH}(μ-N_2_BC_8_H_14_)(μ-H)_2_{Ta(NPN)}] via hydroboration of the Ta–N_2_ unit [31,32]. The resulting boryl–N_2_ complex subsequently underwent H_2_ reductive elimination, N–N bond cleavage, and ligand arrangement, yielding the borylimide–nitride complex. This difference is presumably due to the great tendency of **2-Na** to reductively eliminate H_2_.

The 9-BBN-induced formation of **2-Na** from **3-Na** is closely related to the reaction of [{(PNP)Ti}_2_(μ-η^1^:η^2^-N_2_)(η-H)_2_] with pinacol borane (HBpin), wherein the binding of the borane center to the coordinated N_2_ unit was proposed to trigger reductive elimination of H_2_ and N–N bond cleavage [16]. In addition, the nitride bridged titanium dimer was shown to react with HBpin, yielding the borylimide complex. Mézailles also reported that the N_2_-derived nitride complex [(PPP)Mo(N)I] (PPP = PhP(CH_2_CH_2_PCy_2_)_2_) reacted with HBpin to afford borylamines via stepwise functionalization at the nitride group [33]. Therefore, it was of interest to investigate the possibility of hydroboration of the bridging nitride ligands in **2-Na**.

**Figure 2:**
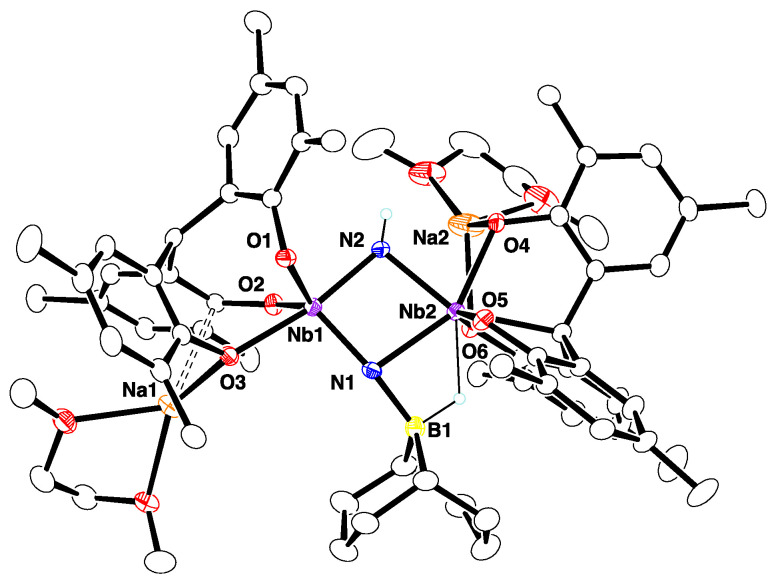
Molecular structure of **4** with thermal ellipsoids set at 30% probability level. All hydrogen atoms on carbon and methyl groups of *tert*-butyl groups are omitted for clarity.

On the NMR tube scale, no reaction was observed at room temperature between a 1:1 mixture of **2-Na** and 9-BBN in C_6_D_6_. Heating at 80 °C for 3 days led to the consumption of all of the 9-BBN and the formation of [{Na(dme)}_2_{(O_3_)Nb}(μ-NH)(μ-NBC_8_H_14_){Nb(O_3_C)}] (**4**) (Scheme 1). However, a small amount of **2-Na** remained unreacted because 9-BBN partially decomposed during the reaction. Therefore, further addition of 9-BBN gave complete conversion to **4**. The reaction was scaled up using excess 9-BBN to give **4** in a 62% isolated yield. The X-ray crystal structure of **4** (Figure 2) confirmed its composition and revealed that the product was the result of an N–B and N–H bond formation and a [O_3_] methine C–H bond scission.

The diniobium complex **4** is *C*_1_ symmetric. The original [O_3_] ligand, which was bound to the Nb2 atom, was transformed into a tetradentate [O_3_C] ligand via C–H activation of the bridgehead methine, while the other [O_3_] ligand bound to the Nb1 atom remained intact. The two Nb atoms were bridged by a parent imide and a 9-BBN-bound nitride ligand, with bond lengths being Nb1–N1 = 1.855(2), Nb1–N2 = 1.942(2), Nb2–N1 = 2.202(2), and Nb2–N2 = 2.111(2) Å. A 9-BBN unit was attached to a nitride group and exhibited a B1–H1–Nb1 agnostic interaction. The B1–N1 bond length of 1.541(4) Å was in the range expected for an N→B dative bond [34,35]. These geometrical parameters are consistent with the bonding sequence shown in Scheme 1. The central Nb_2_N_2_ core of **4** is structurally related to the motif formed via the hydroboration of coordinated N_2_ in [{(NPN)Ta}_2_(μ-η^1^:η^2^-N_2_)(μ-H)_2_] [31,32]. Attempts to observe and isolate any intermediates in the conversion of **2-Na** to **4** were unsuccessful, preventing elucidation of the mechanism of the reaction. The source of the NH proton remains to be determined.

The ^1^H NMR spectrum of **4** indicates a highly asymmetric compound, with twelve inequivalent *tert*-butyl singlets and one methine singlet (5.82 ppm). The N–H resonance was observed at 10.6 ppm and split into a doublet (^1^*J*_NH_ = 72.2 Hz) when the ^15^N-labeled complex was used. The B–H resonance could not be clearly assigned due to overlap with resonances from the BC_8_H_14_ group. The ^11^B NMR spectrum contained a resonance at 50.3 ppm. The ^15^N NMR spectrum of the ^15^N-labeled complex displayed 374 and 445 ppm, which were shifted upfield in comparison to that of **2-Na-^15^N** at 680 ppm. The resonance at 374 ppm was assigned to the NH ligand, but the large quadruple moment of the ^93^Nb nucleus prevented observation of the N–H scalar coupling.

### 2.2. Reactions with ZnMe_2_ and AlMe_3_

**Scheme 2:**
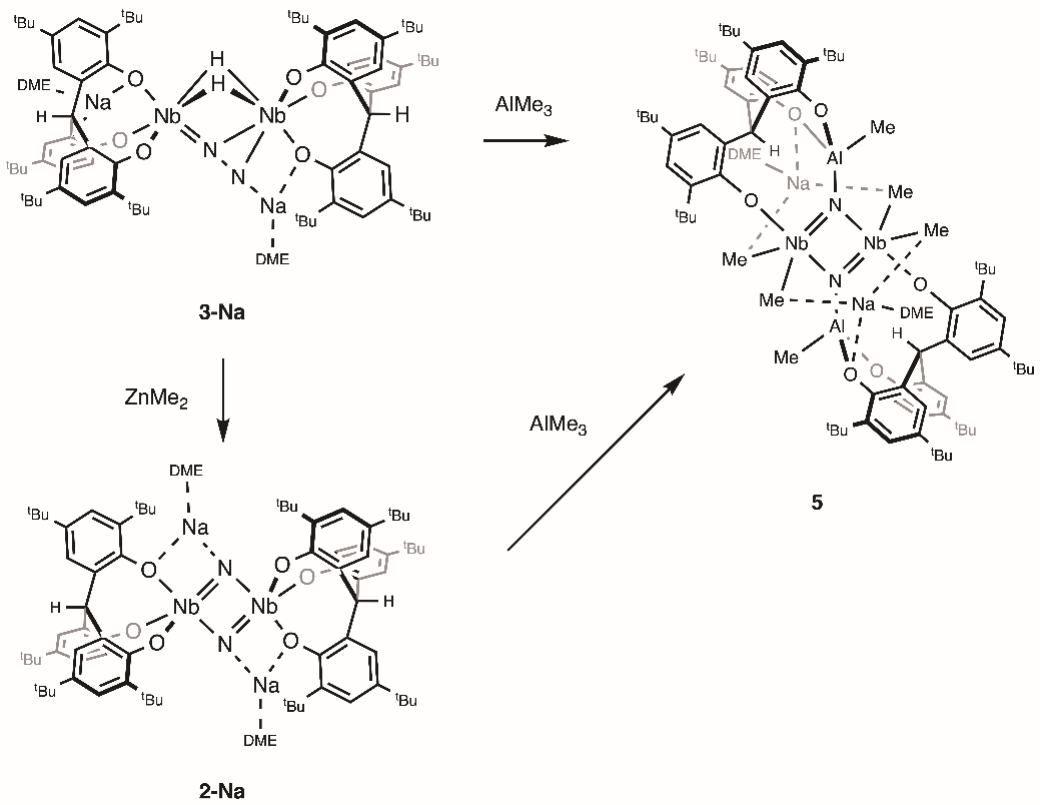
Reactions of **3-Na** with ZnMe_2_ and AlMe_3_.

The borane 9-BBN mediates the conversion of **3-Na** to **2-Na** by behaving as a Lewis acid. Prior to reductive elimination of H_2_, the reaction may proceed through a putative borane adduct. The exact role of 9-BBN is unclear, because the interaction may occur at the coordinated N_2_ unit, the phenoxide groups, or the hydride ligands. Aiming to isolate acid–base adducts and confirm where the interaction occurs, we studied the behavior of the side-on end-on N_2_ complex **3-Na** with other Lewis acids, ZnMe_2_ and AlMe_3_ (Scheme 2). However, we found that these Lewis acids also promote N–N bond cleavage of **3-Na**.

Addition of ZnMe_2_ to **3-Na** in toluene at room temperature resulted in an immediate color change from dark green to brown and the formation of a mixture of products. The products contained **2-Na** according to ^1^H NMR spectroscopy, but they proved to be inseparable upon workup. In contrast to the reaction with 9-BBN, ZnMe_2_ was consumed during the reaction. The formation of CH_4_ was observed but attempts to identify any product containing Zn were unsuccessful.

When two equivalents of AlMe_3_ were added to a solution of **2-Na** in toluene, the reaction mixture immediately turned brown with the concomitant liberation of H_2_. Workup of the reaction mixture led to the isolation of [{Na(dme)}_2_{(O_3_)AlMe}_2_(NbMe_2_)_2_(μ-N)_2_] (**5**) as a brown powder in 69%. The complex **5** was also prepared by the reaction of **3-Na** with two equivalents of AlMe_3_ in toluene at room temperature. This suggests that the reaction of **3-Na** with AlMe_3_ initially yields the nitride complex **2-Na** in a manner analogous to the reaction with 9-BBN. The nitride complex **2-Na** would then form the Lewis acid–base adducts with AlMe_3_, which would undergo further arrangements to produce **5**. The potassium salt was also prepared by the analogous reaction of **2-K** with AlMe_3_.

**Figure 3:**
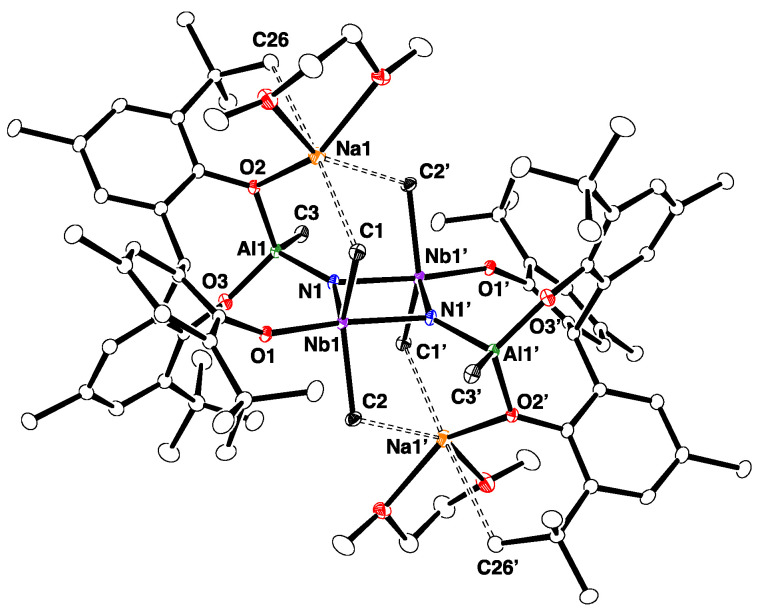
Molecular structure of **5** with thermal ellipsoids set at 30% probability level. All hydrogen atoms and methyl groups of *para*-*tert*-butyl substitutes of aryloxide are omitted for clarity.

The X-ray crystal structure of **5** (Figure 3) revealed that the molecule lies on a crystallographic inversion center in the middle of the Nb_2_N_2_ core. Each niobium atom adopts a trigonal bipyramidal geometry, with two methyl groups, two nitride groups, and one phenoxide group of the [O_3_] ligand. The complex **5** contains two Al atoms, each of which is tetrahedrally coordinated by two phenoxide groups of the [O_3_] ligand, one methyl group, and one nitride group. The Nb_2_N_2_Al_2_ core closely approaches planarity, imparting a trigonal plane geometry to each nitrogen atom. Two solvated sodium cations are retained as a part of a cluster structure, each being bridged to the Nb_2_Al core by two methyl groups and one phenoxide group. The Nb–N bond lengths of 1.8719(13) and 2.0870(13) Å are comparable with those of **2-Na**. The Al–N bond length of 1.8980(14) Å is longer than those for Al–N(amide) bonds [36,37] and shorter than those for Al–N dative bonds [38,39].

The ^1^H NMR spectrum of **5** shows four singlets for the *tert*-butyl groups in a 1:1:2:2 ratio, which is consistent with the average *C*_2h_ symmetry in the molecule. The methine protons of the [O_3_] ligand appear as a singlet at 7.00 ppm. The two singlets for the Nb–Me and Al–Me groups are observed at 1.46 and −0.02 ppm in a 2:1 ratio, respectively. The ^15^N NMR spectrum of the ^15^N-labeled complex displays a single resonance upfield shifted to 553 ppm, indicative of coordination to aluminum.

## 3. Conclusions

We demonstrated that the side-on end-on dinitrogen hydride complex, **3-Na**, readily underwent reductive elimination of H_2_ in the presence of Lewis acids such as 9-BBN, AlMe_3_, and ZnMe_2_, followed by N–N bond cleavage to produce **2-Na**. The reactions are proposed to proceed via initial formation of Lewis acid adducts with **3-Na** at the β-N atom of the coordinated dinitrogen, taking into account the nucleophilicity of the β-N atom of the side-on end-on N_2_ unit and the silylation of the β-N atom by the reaction of **3-Na** with Me_3_SiCl [24]. However, attempts to observe and isolate any adducts failed, because the Lewis acid adducts of **3-Na** are quite prone to reductive elimination of H_2_. When other Lewis acids such as HBpin and B(C_6_F_5_)_3_ were used, the reactions yielded intractable mixtures. The resulting nitride complex, **2-Na**, was found to further react with 9-BBN and AlMe_3_ to produce **4** and **5**, respectively. Further work will focus on the functionalization of the coordinated N_2_ unit in **3-Na**.

## 4. Materials and Methods

### 4.1. General Information

All manipulations were carried out using standard Schlenk techniques or in a glovebox under an atmosphere of argon. Anhydrous hexane, pentane, and toluene were dried by passage through two columns of activated alumina and a Q-5 column, while anhydrous THF, Et_2_O, and DME were dried by passage through two columns of activated alumina. Anhydrous deuterated benzene (benzene-*d*_6_) was dried and degassed over a potassium mirror prior to use. NMR spectra were recorded on a JEOL ECX-500 spectrometer (JEOL Ltd., Tokyo, Japan). ^1^H NMR spectra were reported with reference to solvent resonances of C_6_D_6_ residual protons (δ = 7.16 ppm). ^13^C NMR spectra were referenced to solvent (peaks δ = 128.06 (C_6_D_6_) ppm). ^11^B chemical shifts were referenced to BF_3_•OEt_2_ (neat at 0.0 ppm) as an external standard. ^15^N chemical shifts were referenced to 90% formamide in dimethyl sulfoxide-*d*_6_ (112.7 ppm with respect to NH_3_ at 0.0 ppm) as an external standard. Complexes **2-K** and **3-Na** were prepared following the literature procedure [23,24]. Elemental analyses (C, H, and N) were carried out on an Elementar vario MICRO Cube (Elementar, Frankfurt, Germany). IR spectra were recorded on a JASCO VIR-200 spectrometer (JASCO, Tokyo, Japan). Spectra and structures are available in Supplementary Materials.

### 4.2. Synthesis of **2-Na**

To a solution of **3-Na** (170 mg, 0.100 mmol) in toluene (6 mL) was added 9-BBN (25.0 mg, 0.205 mmol). The solution was stirred at room temperature overnight and changed color from green to brown. The volatile materials were removed under vacuum. The residue was washed with hexane and was then dried under vacuum, leaving a brown powder of **2-Na** (67% yield, 114 mg). ^1^H NMR (C_6_D_6_, 400 MHz, 323 K): δ 1.23 (s, 54H, ^t^Bu), 1.69 (s, 54H, ^t^Bu), 2.83 (s, 12H, DME), 2.86 (s, 8H, DME), 5.66 (s, 2H, CH), 7.14 (d, *J* = 2.6 Hz, 6H, ArH), and 7.37 (d, *J* = 2.6 Hz, 6H, ArH). ^13^C NMR (C_6_D_6_, 125.8 MHz): δ 31.5, 31.8, 34.3, 36.0 (^t^Bu), 59.5 (DME), 60.1 (CH), 70.3 (DME), 122.5, 125.7, 138.8, 140.5, and 161.3. One peak for the aromatic carbon was not assigned, probably due to overlapping. Calcd for C_94_H_142_N_2_Na_2_Nb_2_O_10_: C, 66.73; H, 8.46; N, 1.66. Found: C, 66.44; H, 8.38; N 0.75.

### 4.3. Synthesis of **2-Na-^15^N**

The ^15^N-labeled analogue was prepared in a manner identical to that used for **2-Na**, except for using the ^15^N-labeled precursor **3-Na-^15^N**. ^15^N NMR (C_6_H_6_, 50.7 MHz): δ 679.1.

### 4.4. Synthesis of **4**

To a solution of **3-Na** (42.4 mg, 25.0 μmol) in toluene (6 mL) was added 9-BBN (12.2 mg, 100 μmol). The solution was stirred at room temperature overnight and was then allowed to warm to 80 °C. The color of the solution turned from green to brown to orange. After stirring for 2 days, the volatile materials were removed under vacuum. The residue was washed with hexane, leaving **4** as a yellow powder (62%, 28.0 mg). ^1^H NMR (C_6_D_6_, 400 MHz): δ 1.20, 1.21, 1.24, 1.36, 1.39, 1.50, 1.56, 1.62, 1.66, 1.67, 1.69, 1.71 (s, 9H each, ^t^Bu), 0.93–3.84 (complicated overlapping resonances, 14H total, BC_8_H_14_), 2.40 (br, 12H, DME), 2.53 (br, 8H, DME), 5.82 (s, 1H, CH), 7.06, 7.10, 7.18, 7.23, 7.30, 7.32, 7.36, 7.43, 7.63, 7.66, 8.45, 8.53 (d, *J* = 2 Hz, 1H each, ArH), 10.57 (s, 1H, NH). ^13^C NMR (C_6_D_6_, 125.8 MHz): δ 31.2, 31.5, 31.6 (2C), 31.7, 31.95, 31.99, 32.1, 32.3, 32.4, 32.47, 32.53, 33.7 (2C), 34.15, 34.21, 34.5 (2C), 34.8, 34.9, 35.3, 35.6, 36.0, 36.3 (^t^Bu), 25.1, 25.9 (2C), 28.0, 33.5, 36.1, 39.9 (9–BBN), 58.9 (CH), 58.8, 70.2 (DME), 119.7, 120.1, 120.4, 122.0, 122.2, 122.7, 122.8, 124.3, 124.9, 125.8, 126.6, 129.0, 130.7, 132.1, 133.7, 135.1, 135.9, 138.2, 140.1, 140.16, 140.24, 140.9, 141.1, 141.2, 142.0, 142.8, 143.7, 147.2, 148.5, 149.7, 159.1, 160.2, 160.9, 163.6, 164.0, 164.8. One peak for 9-BBN was not assigned, probably due to overlapping. ^11^B NMR (C_6_D_6_, 160.5 MHz) δ 50.3. Anal. Calcd for C_102_H_157_BN_2_Na_2_Nb_2_O_10_: C, 67.54; H, 8.72; N, 1.54. Found: C, 67.10; H, 8.55; N, 1.57. IR (KBr; *ν*/cm^–1^) 3447 (N–H).

### 4.5. Reaction of **2-Na** with 9-BBN

To a solution of **2-Na** (15.2 mg, 8.98 μmol) in C_6_D_6_ (0.6 mL) was added 9-BBN (1.1 mg, 9.0 μmol). The ^1^H NMR spectrum was identical to that of a sample of isolated **4**.

### 4.6. Reaction of **2-K-^15^N** with 9-BBN

To a solution of **2-K** (14.5 mg, 8.40 μmol) in C_6_D_6_ (0.6 mL) was added 9-BBN (1.0 mg, 8.2 μmol). The ^1^H NMR spectrum was similar to that of a sample of isolated **4**, except for a resonance attributed to the N–H group. ^1^H NMR (C_6_D_6_, 500.2 MHz): 1.19, 1.22, 1.30, 1.40, 1.43, 1.46, 1.56, 1.60, 1.67, 1.71, 1.74, 1.75 (s, 9H each, ^t^Bu), 0.94–3.76 (complicated overlapping resonances, 14H total, BC_8_H_14_), 2.66 (s, 15H, DME), 2.72 (s, 10H, DME), 5.83 (s, 1H), 7.06, 7.22, 7.24, 7.39, 7.40, 7.47, 7.57, 7.61, 8.36, 8.57 (br, 1H each, ArH) 10.96 (d, ^1^*J*_NH_ = 72.2 Hz, 1H, NH). Two peak for Ar–H were not assigned, probably due to overlapping. ^11^B NMR (C_6_D_6_, 160.5 MHz,); δ 60.8. ^15^N NMR (C_6_D_6_, 50.7 MHz): δ 374.4 (NH), 444.7 (NB).

### 4.7. Reaction of **3-Na** with ZnMe_2_

To a solution of **3-Na** (16.8 mg, 9.92 μmol) in C_6_D_6_ (0.6 mL) was added ZnMe_2_ (1.05 M in hexane, 10.0 μL, 10.5 μmol). The ^1^H NMR spectrum contained resonances identical to that of a sample of isolated **2-Na**.

### 4.8. Synthesis of **5**

To a solution of **3-Na** (104 mg, 61.5 μmol) in toluene (4 mL) was added a solution of AlMe_3_ (1.05 M in hexane, 117 μL, 123 μmol) at room temperature. The solution turned brown instantly and was stirred overnight. The volatile materials were removed under vacuum, and then the residue was washed with hexane. The product **5** was isolated as an orange solid (76.8 mg, 41.8 μmol, 69%). ^1^H NMR (C_6_D_6_, 500.2 MHz): δ −0.02 (s, 6H, AlCH_3_), 1.32 (s, 36H, ^t^Bu), 1.46 (s, 12H, NbCH_3_), 1.50 (s, 18H, ^t^Bu), 1.59 (s, 36H, ^t^Bu), 1.67 (s, 18H, ^t^Bu), 2.39 (br, 12H, DME), 2.55 (br, 8H, DME), 7.00 (s, 2H, CH), 7.28 (d, *J* = 2.6 Hz, 4H, ArH), 7.40 (br, 4H, ArH), 7.49 (d, *J* = 2.5 Hz, 2H, ArH), 8.23 (br, 2H, ArH). Anal. Calcd for C_108_H_180_Al_2_N_2_Na_2_Nb_2_O_14_: C, 64.33; H, 9.00; N, 1.39. Found: C, 64.61; H, 9.28; N, 1.59. The low solubility of **5** in solvent prevented us from acquiring ^13^C NMR spectra.

### 4.9. Reaction of **2-Na** with AlMe_3_

To a solution of **2-Na** (16.8 mg, 9.93 μmol) in toluene (2 mL) was added AlMe_3_ (1.05 M in hexane, 20 μL, 21 μmol). The ^1^H NMR spectrum was identical to that of a sample of isolated **5**.

### 4.10. Reaction of **2-K-^15^N** with AlMe_3_

To a solution of **2-K-^15^N** (10.3 mg, 5.97 μmol) in C_6_D_6_ (0.6 mL) was added AlMe_3_ (1.05 M in hexane, 11 μL mg, 11.6 μmol). The ^1^H NMR spectrum was identical to that of a sample of isolated **5**. ^15^N NMR (C_6_D_6_, 50.7 MHz): δ 552.9.

### 4.11. X-ray Crystallography

Single crystals were immersed in immersion oil on a micromount and transferred to a Rigaku Varimax with a Saturn system or a Rigaku XtaLAB Synergy-DW system equipped with a Rigaku GNNP low temperature device (Tokyo, Japan). Data were collected under a cold nitrogen stream at 123 K or 173 K using graphite-monochromated MoKα (λ = 0.71073 Å) or CuKα (λ = 1.54184 Å) radiation. Equivalent reflections were merged, and the images were processed with the CrysAlis^Pro^ software 1.171.42.64a (Rigaku Oxford Diffraction, Japan). Empirical absorption corrections were applied. All structures were solved by direct method using SHELXT [40] and refined by full-matrix least-squares method on *F*^2^ for all data using SHELXL [41] with the Olex2 program [42]. All hydrogen atoms were placed at their geometrically calculated positions. For **4**, the hydrogen atoms of the NH group and the BH group were located in the Fourier map and refined isotropically. For **2-Na** and **4**, some residual electron density was difficult to model, the program SQUEEZE [43] was used to remove the contribution of the electron density in the solvent region from the intensity data. For **2-Na**, one *tert*-butyl group was disordered. A void space contains 175 electrons per unit cell, which could be attributed to distorted pentane molecules (one molecule in the asymmetric unit). A large residual peak in the final difference map was located in the Nb_2_N_2_ core. This peak is believed to be due to a small amount of twinning in the crystal. For **4**, two *tert*-butyl groups and one DME molecule were disordered. For **4**, a void space contains 1013 electrons per unit cell, which could be attributed to distorted pentane molecules (three molecules in the asymmetric unit). For **5**, one benzene molecule was disordered.

## Data Availability

The original data presented in this study are available from the authors.

## Supplementary Materials

The following supporting information can be downloaded at: https://www.mdpi.com/article/10.3390/molecules27175553/s1, NMR spectra (Figures S1–S11), crystallographic details (Table S1), (Figures S12–S14), structures and IR spectra (Figures S15–S17) for complexes **2-Na**, **4**, and **5**. The CCDC deposition numbers 2195593–2195595 contain the supplementary crystallographic data for this paper, which can obtained free of charge via emailing data_request@ccdc.cam.ac.uk, or by contacting The Cambridge Crystallographic Data Centre at 12 Union Road, Cambridge CB2 1EZ, UK; fax: +44 1223 336033.

## Sample Availability

Not available.
