# Supplementary material for: Lewis Acid-Induced Dinitrogen Cleavage in an Anionic Side-on End-on Bound Dinitrogen Diniobium Hydride Complex
Source: Molecules. 2022 Aug 29;27(17):5553. doi: 10.3390/molecules27175553 (PMC9457992; doi:10.3390/molecules27175553)
Supplement: Supplementary file 1 [file molecules-27-05553-s001.zip › molecules-1884863-supplementary.pdf]

(Supporting Information)

Lewis Acid-Induced Dinitrogen Cleavage in an Anionic Side-on End-on Bound Dinitrogen Diniobium Hydride Complex

Naofumi Suzuki, Yutaka Ishida, and Hiroyuki Kawaguchi

Department of Chemistry, Tokyo Institute of Technology,

Ookayama, Meguro-ku, Tokyo 152-8551, Japan

Table of Contents

NMR spectra	S2
Molecular structures	S8
Crystallographic data	S11
IR spectra	S13

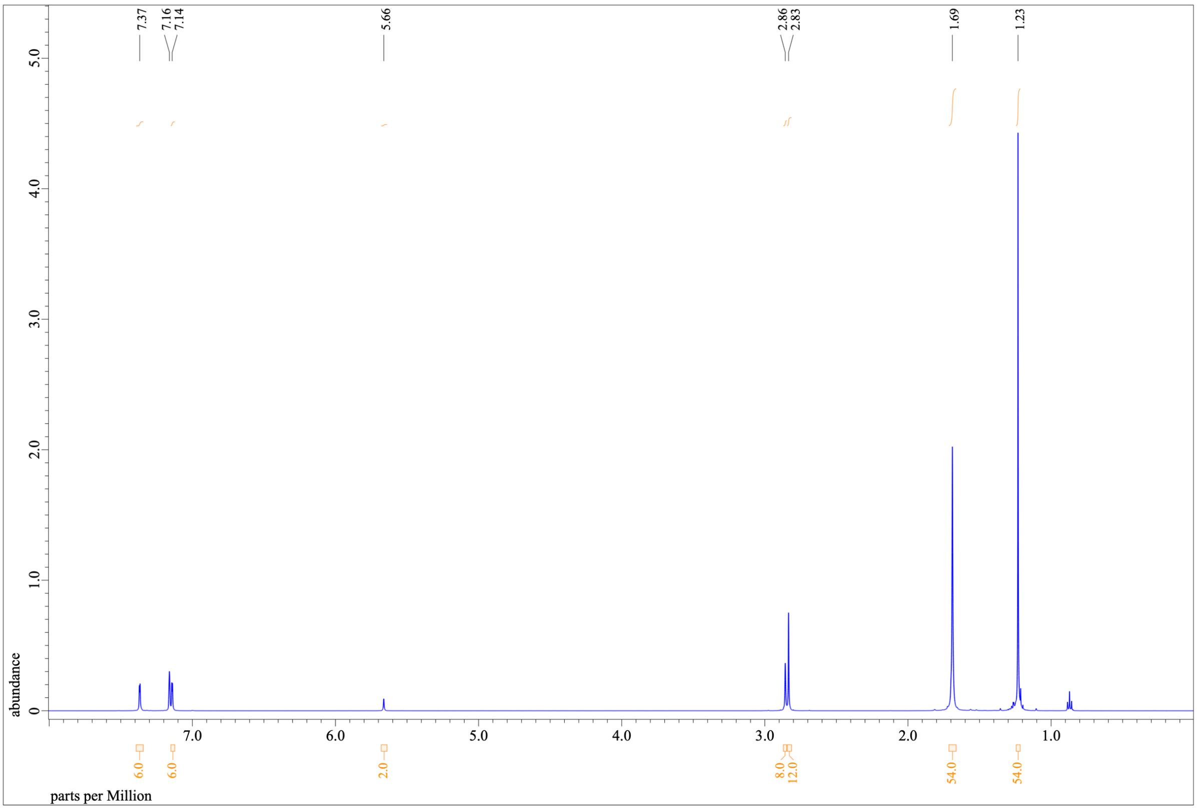

Figure S1. ^{1}H NMR spectrum of **2-Na**.

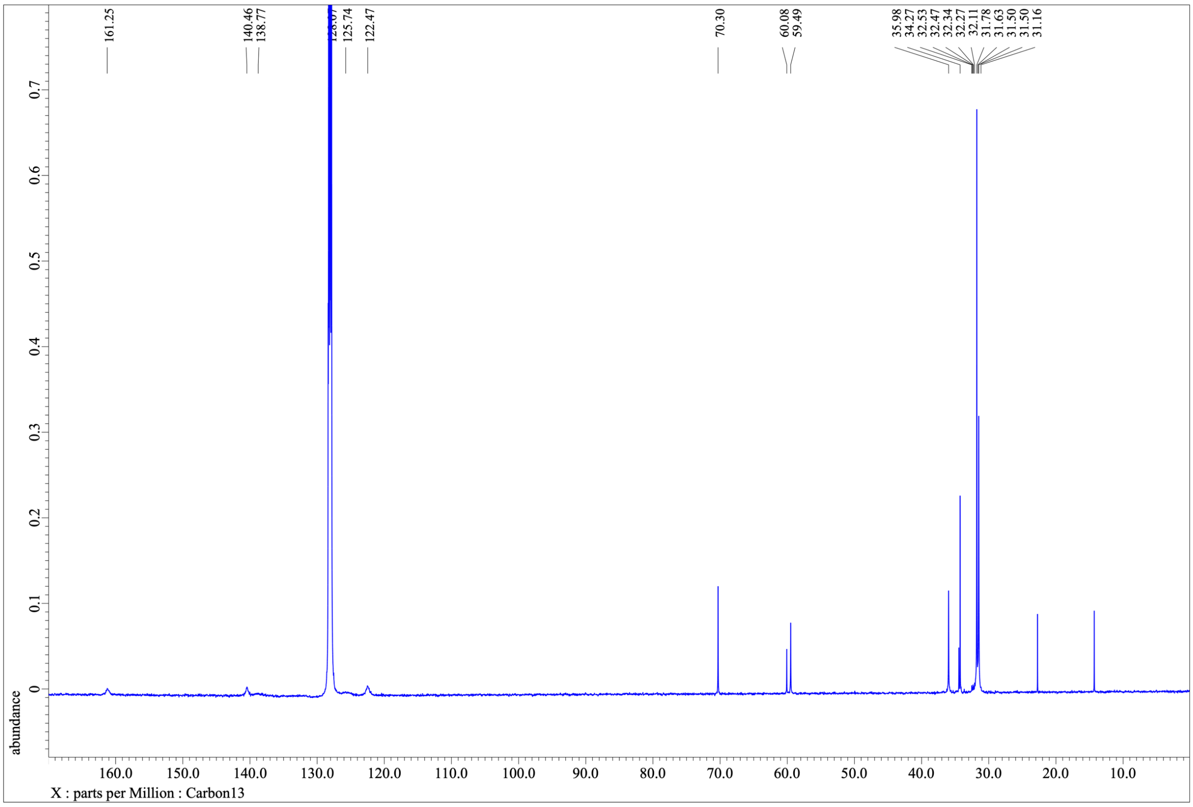

Figure S2. ^{13}C NMR spectrum of **2-Na**.

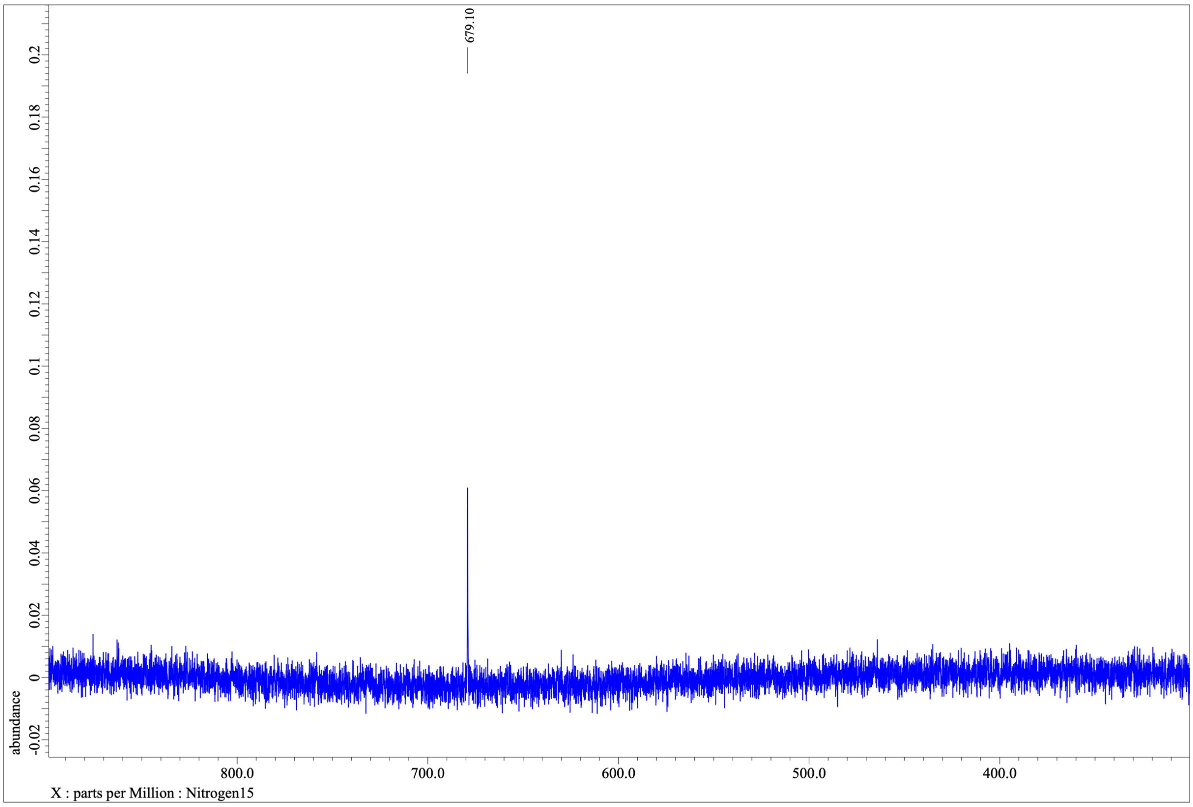

Figure S3. ^{15}N NMR spectrum of **2-Na**.

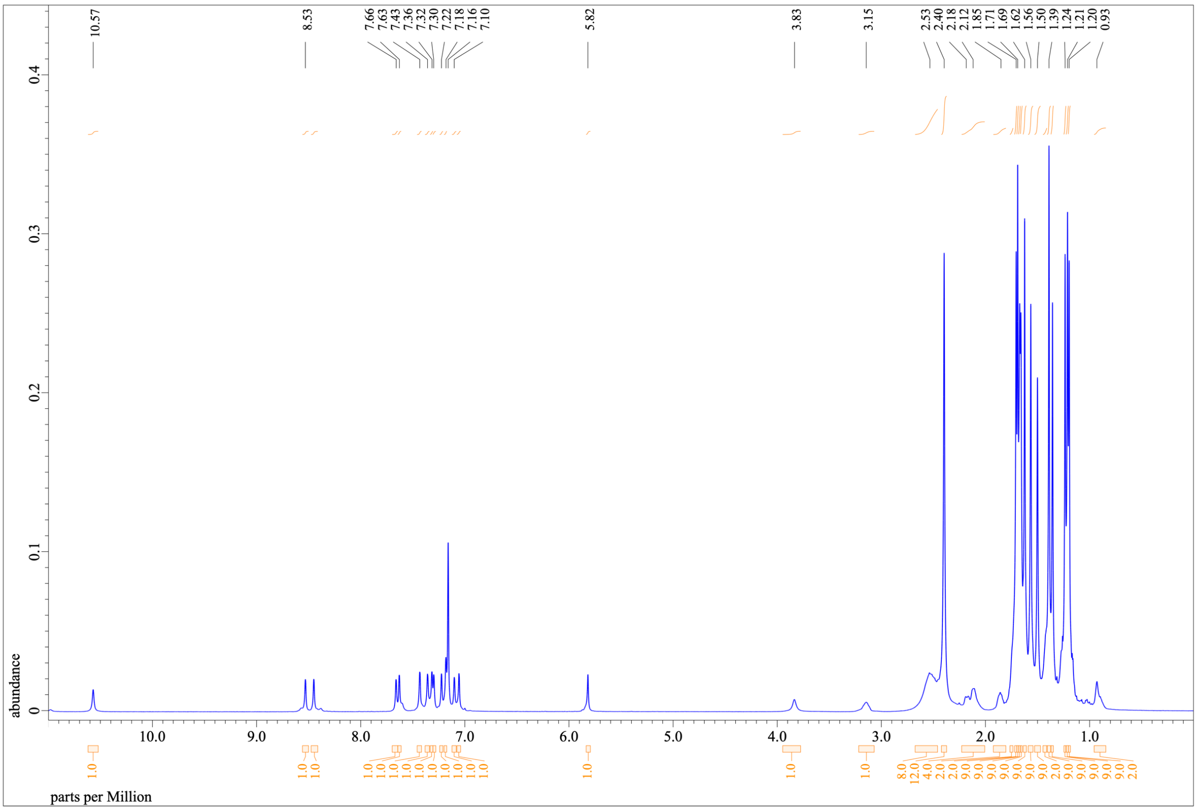

Figure S4. ^{1}H NMR spectrum of **4**.

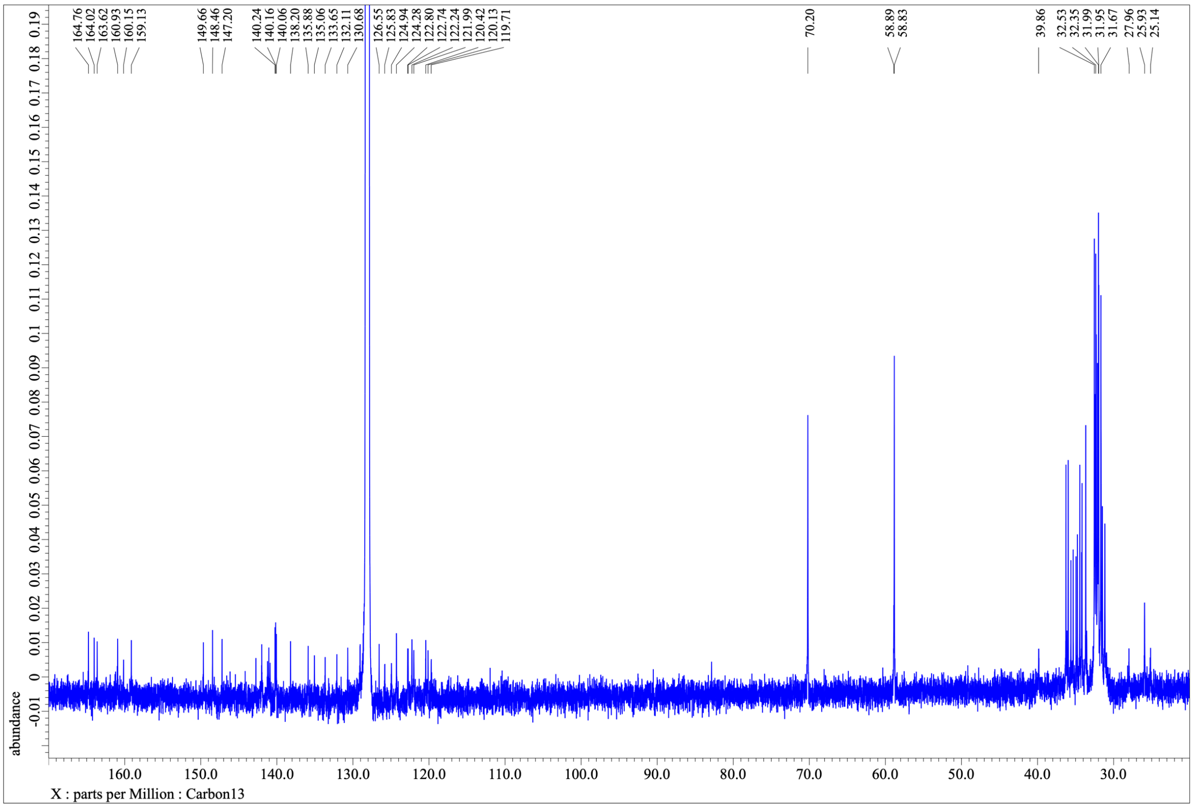

Figure S5. ^{13}C NMR spectrum of **4**.

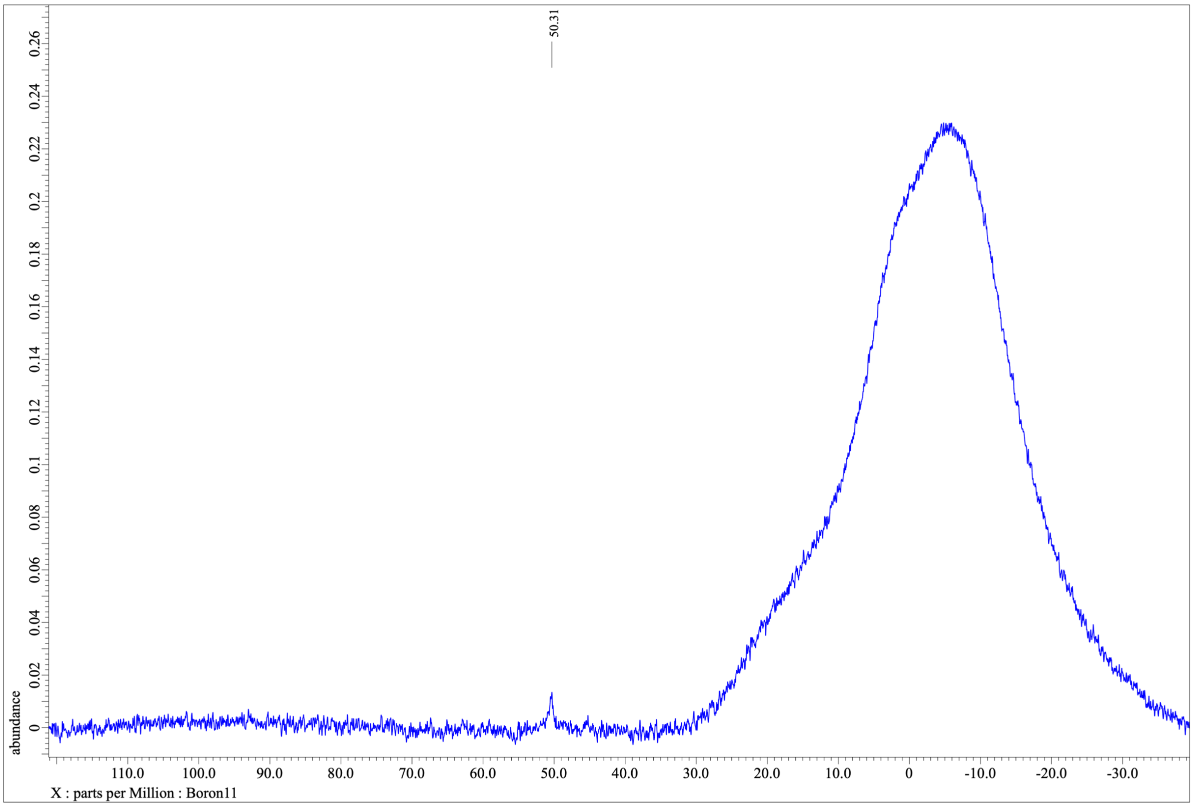

Figure S6. ^{11}B NMR spectrum of **4**.

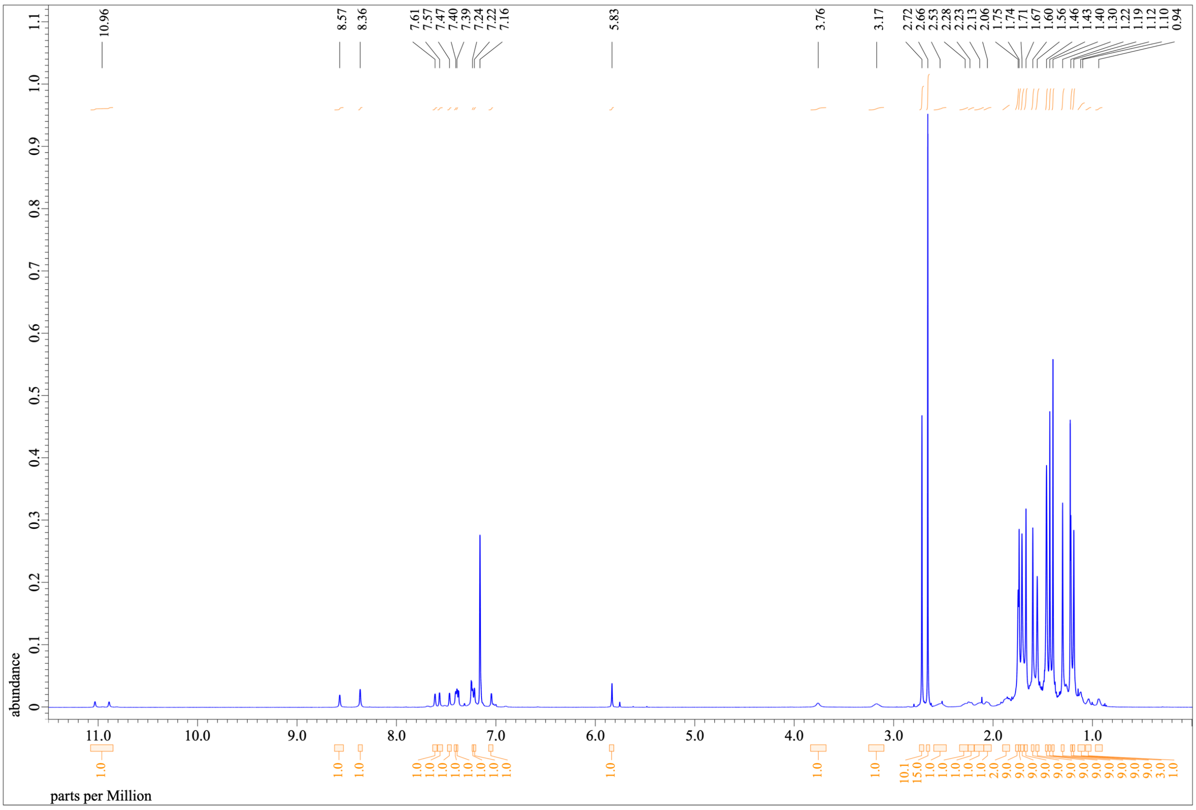

Figure S7. ^{1}H NMR spectrum of **4-K**.

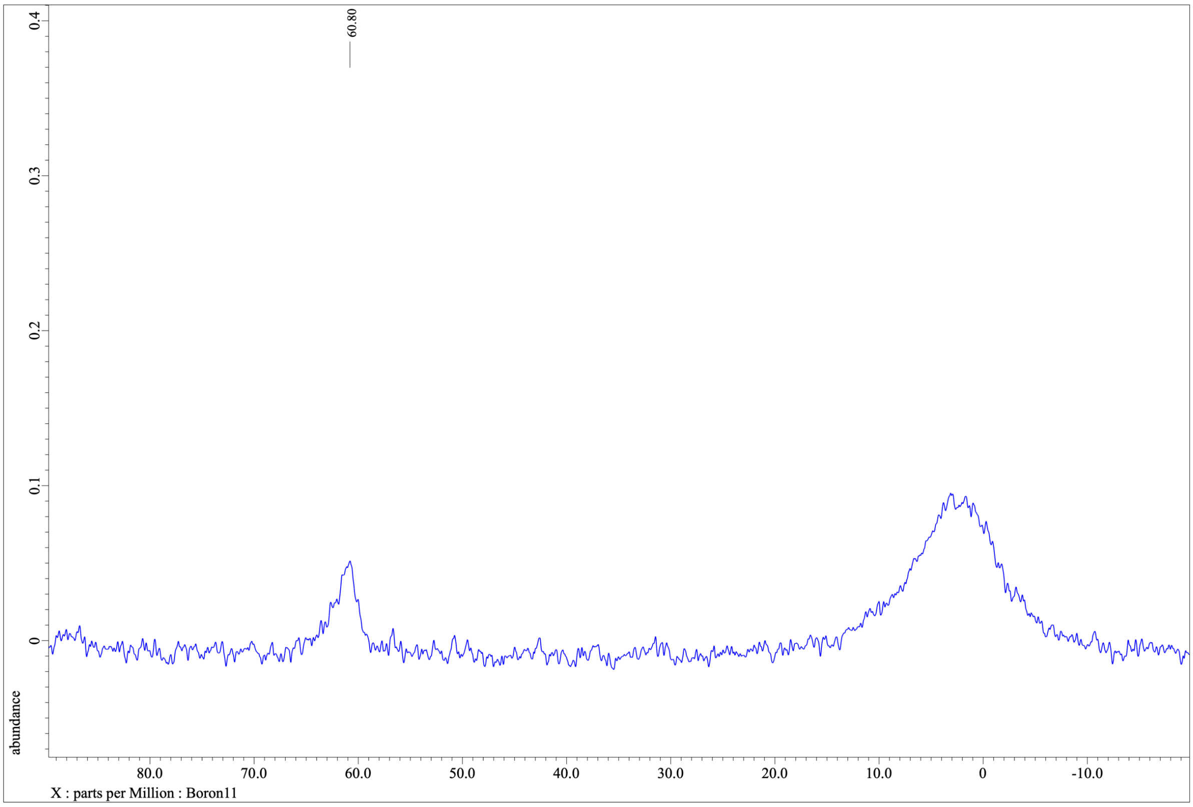

Figure S8. ^{11}B NMR spectrum of **4-K**.

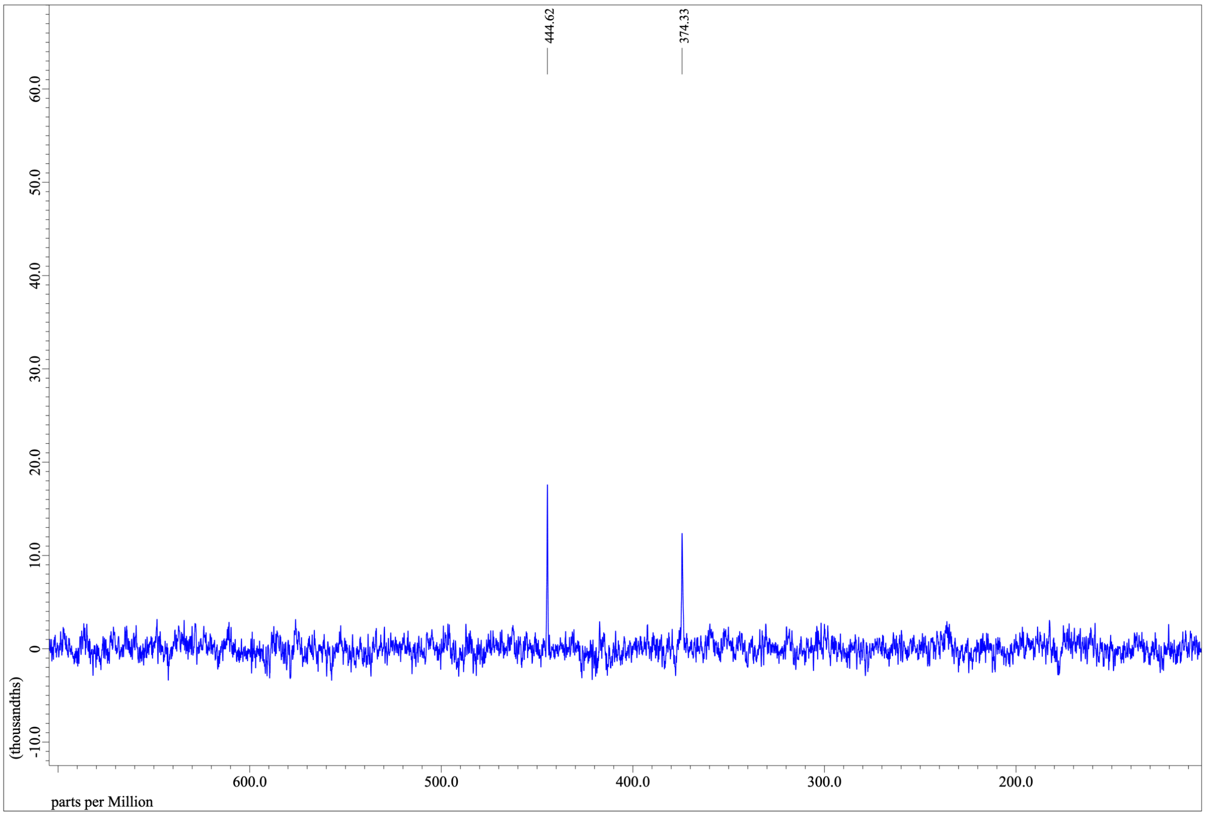

Figure S9. ^{15}N NMR spectrum of **4-K**.

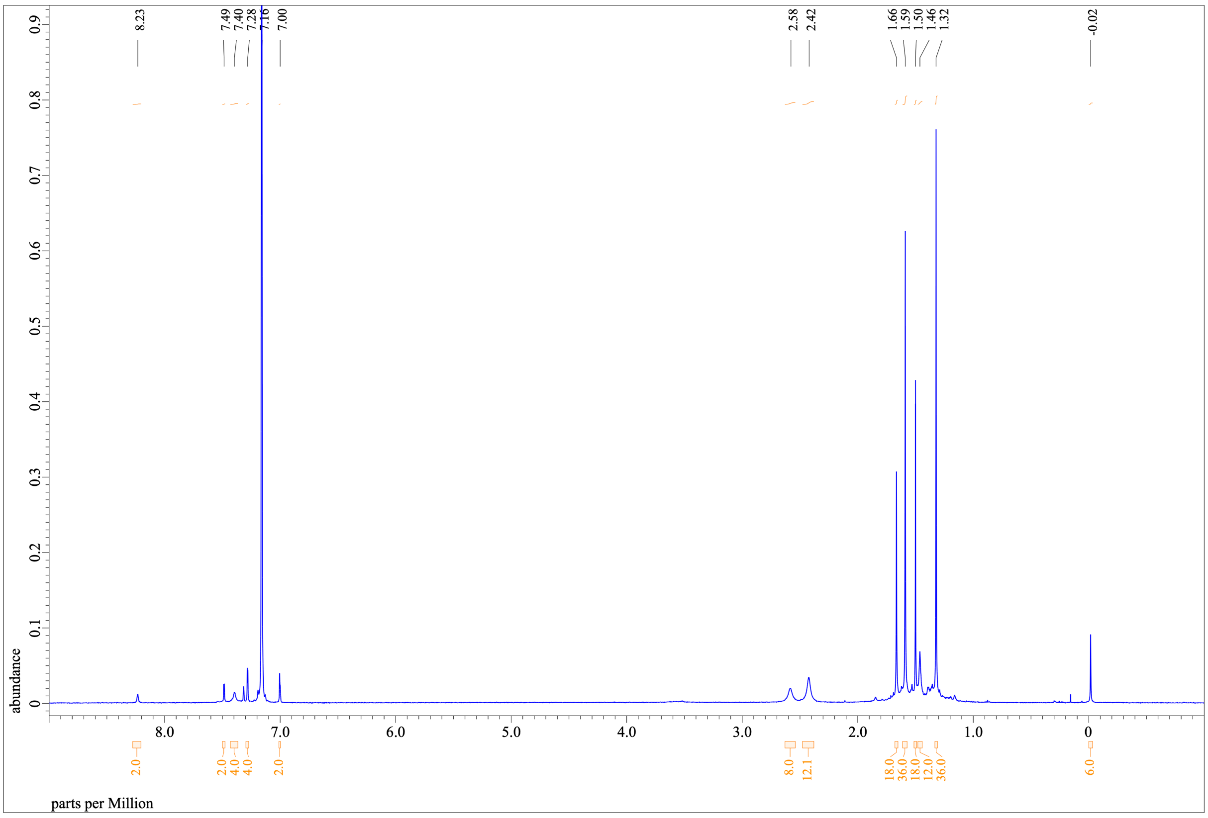

Figure S10. ^{1}H NMR spectrum of **5**.

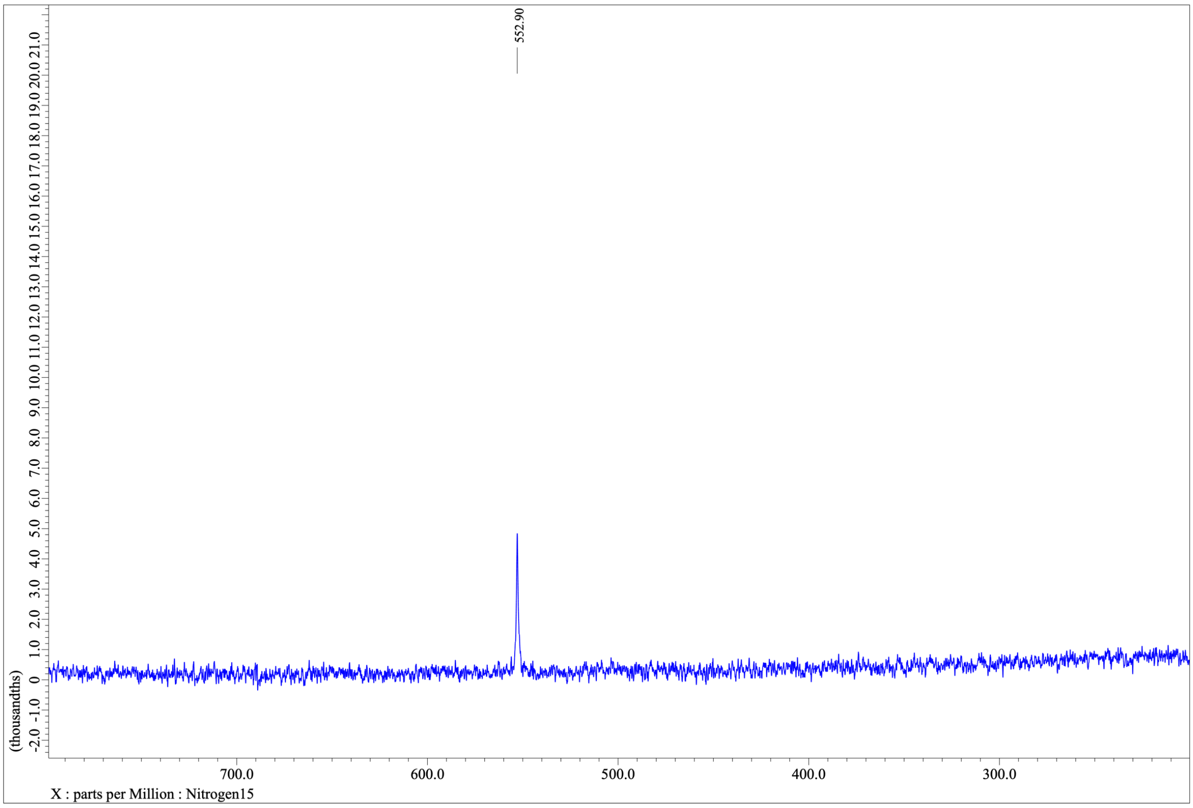

Figure S11. ^{15}N NMR spectrum of **5-K**.

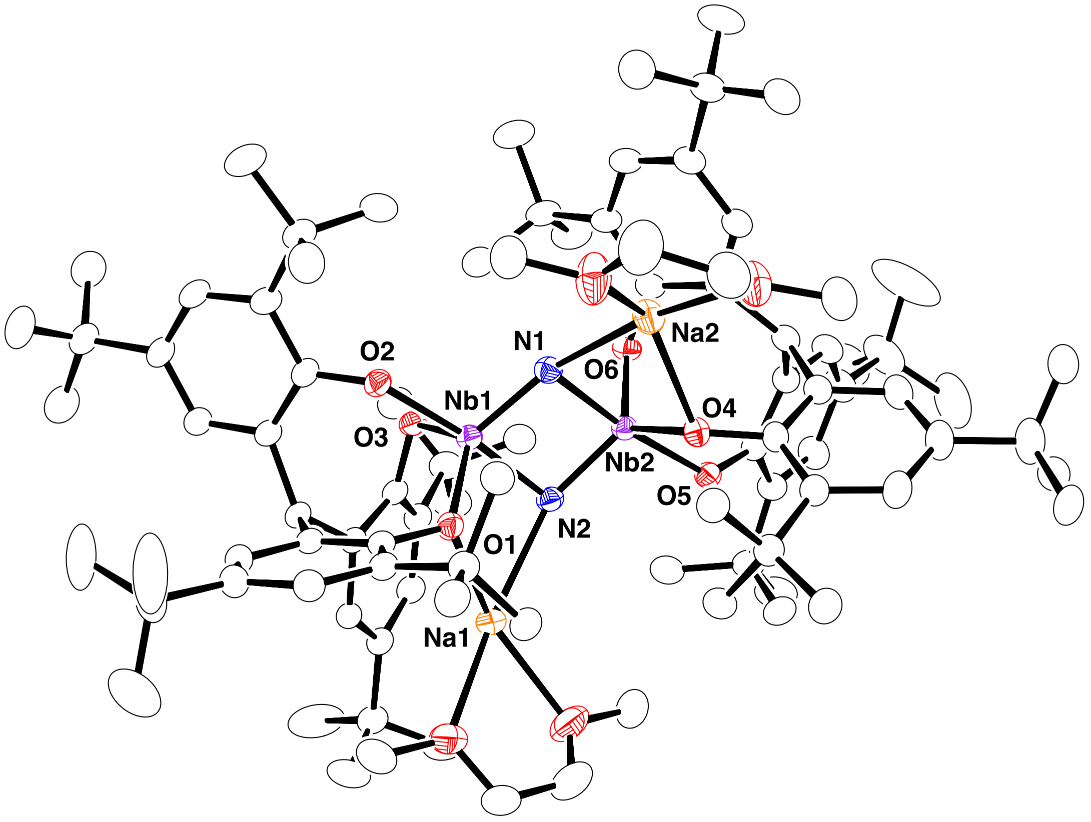

Figure S12. Molecular structure of **2- Na** with thermal ellipsoids set at 30% probability level. All hydrogen atoms are omitted for clarity. Selected bond lengths [Å] and angles [°]: Nb1–O1 2.0236(18), Nb1–O2 2.0321(17), Nb1–O3 2.0146(18), Nb1–N1 1.842(2), Nb1–N2 2.025(2), Nb2–O4 2.0149(17), Nb2–O5 2.0362(17), Nb2–O6 2.0204(17), Nb2–N1 2.027(2), Nb2–N2 1.838(2), Na1–O1 2.392(2), Na1–N2 2.490(2), Na2–O4 2.384(2), Na2–N1 2.451(2), O1–Nb1–O2 86.85(7), O1–Nb1–N2 89.67(8), O3–Nb1–O1 123.89(7), O3–Nb1–O2 84.95(7), O3–Nb1–N2 92.68(8), N1–Nb1–O1 118.42(8), N1–Nb1–O2 101.78(8), N1–Nb1–O3 117.60(8), N1–Nb1–N2 84.48(9), N2–Nb1–O2 173.72(8), O4–Nb2–N1 88.52(8), N2–Nb2–O4 117.39(8), N2–Nb2–N1 84.52(9).

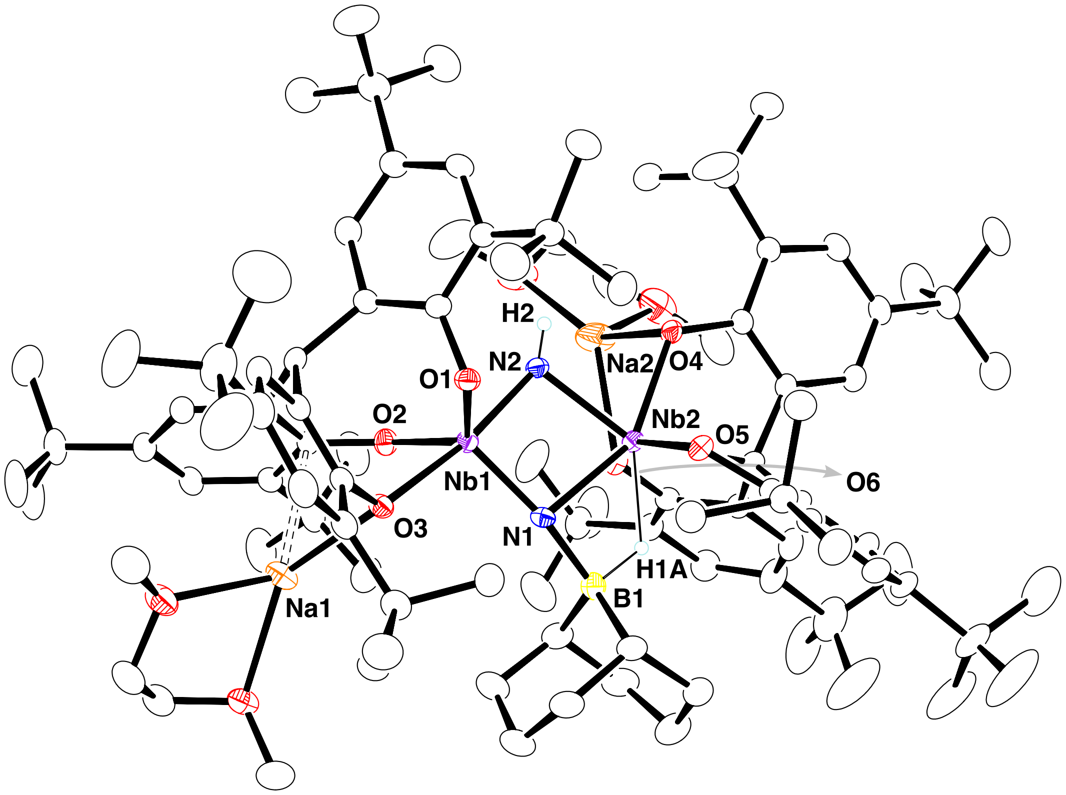

Figure S13. Molecular structure of **4** with thermal ellipsoids set at 30% probability level. All hydrogen atoms on carbon are omitted for clarity. Selected bond lengths [Å] and angles [°]: Nb1–O1 2.0260(19), Nb1–O2 1.9963(19), Nb1–O3 2.1189(18), Nb1–N1 1.855(2), Nb1–N2 1.942(2), Nb2–O4 2.0953(19), Nb2–O5 1.9538(18), Nb2–O6 2.0052(18), Nb2–N1 2.202(2), Nb2–N2 2.111(2), Na1–O3 2.31(2), Na2–O4 2.401(2), N1–B1 1.541(4), Nb2–H1A 1.86(2), N2–H2 0.77(3), B1–H1A 1.14(2). O1–Nb1–O3 78.64(7), O2–Nb1–O1 127.46(8), O2–Nb1–O3 83.16(8), N1–Nb1–O1 117.61(9), N1–Nb1–O2 114.00(9), N1–Nb1–O3 100.59(8), N1–Nb1–N2 88.34(10), N2–Nb1–O1 91.78(9), N2–Nb1–O2 98.95(9), N2–Nb1–O3 169.14(9), O4–Nb2–N1 149.75(8), O4–Nb2–N2 74.54(8), O5–Nb2–O4 93.20(8), O5–Nb2–O6 149.50(8), O5–Nb2–N1 93.29(8), O5–Nb2–N2 112.52(8), O6–Nb2–O4 89.33(8), O6–Nb2–N1 99.68(8), O6–Nb2–N2 97.46(8), N2–Nb2–N1 75.68(9).

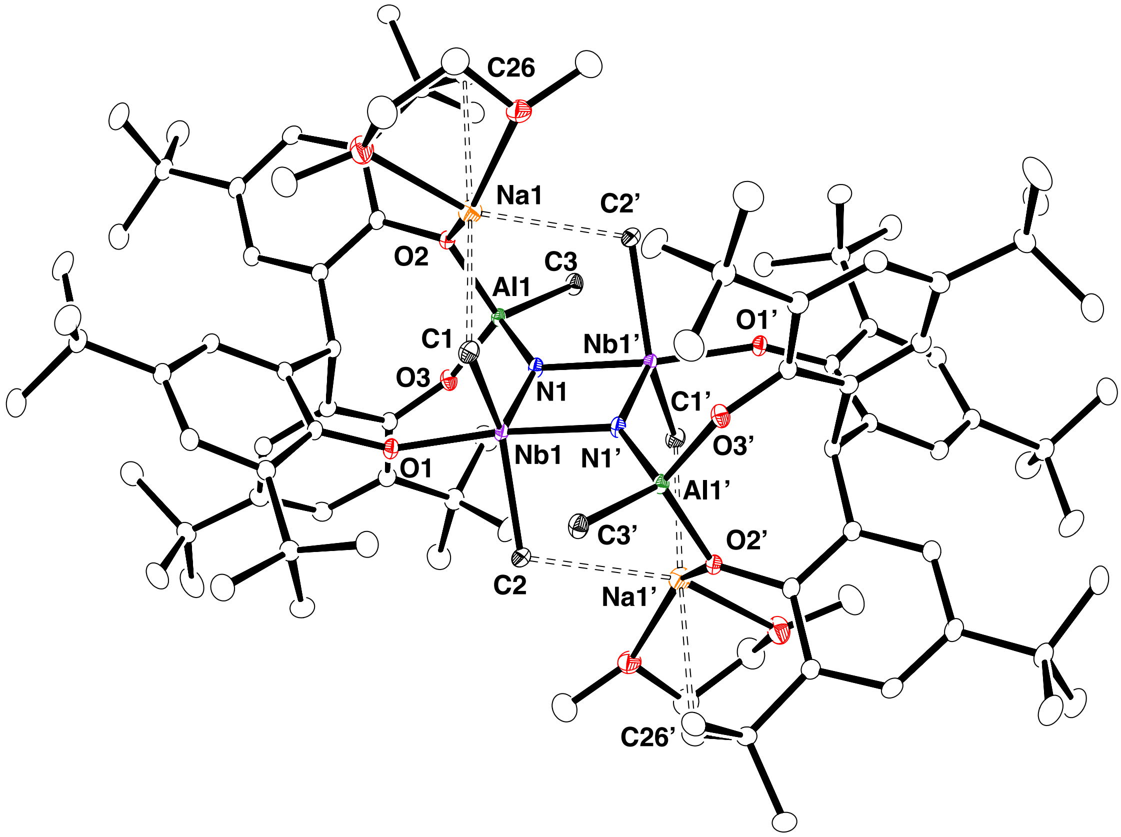

Figure S14. Molecular structure of **5** with thermal ellipsoids set at 30% probability level. All hydrogen atoms are omitted for clarity. Selected bond lengths [Å] and angles [°]: Nb1–Nb1' 2.9678(2), Nb1–O1 1.9413(11), Nb1–N1 1.8719(13), Nb1–N1' 2.0870(13), Nb1–C1 2.2174(16), Nb1–C2 2.2242(16), Al1–O2 1.8042(12), Al1–O3 1.7321(12), Al1–N1 1.8980(14), Al1–C3 1.9567(17), Na1–C1 2.9350(19), Na1–C2’ 2.8804(18), Na1–C26 3.1183(19). O1–Nb1–Nb1' 154.57(3), O1–Nb1–N1' 166.44(5), O1–Nb1–C1 85.30(6), O1–Nb1–C2 89.66(6), N1'–Nb1–Nb1' 38.76(4), N1–Nb1–Nb1' 44.27(4), N1–Nb1–O1 110.37(5), N1–Nb1–N1' 83.03(6), N1–Nb1–C1 105.45(6), N1'–Nb1–C1 89.14(6), N1–Nb1–C2 116.94(6), N1'–Nb1–C2 85.82(6), C1–Nb1–Nb1' 99.06(5), C1–Nb1–C2 136.25(6), C2 – Nb1–Nb1' 103.56(4), O2–Al1–N1 104.01(6), O2–Al1–C3 110.76(7), O3–Al1–O2 115.45(6), O3–Al1–N1 106.77(6), O3–Al1–C3 105.82(7), N1–Al1–C3 114.24(7).

Table S1. Crystallographic data

	**2-Na**	**4**
Formula	$C_{94}H_{14}N_2Na_2Nb_2O_{10}$, C_5H_{12}	$C_{102}H_{157}BN_2Na_2Nb_2O_{10}$, 3 ($C_5H_{12}$)
Formula Mass (g mol^{-1})	1764.03	2030.33
Temperature (*K*)	153	173
Crystal system	*Monoclinic*	*Monoclinic*
Space group	$P2_1/c$ (#14)	$C2/c$ (#15)
Crystal color	Yellow	Yellow
Crystal size (mm)	0.096 × 0.073 × 0.026	0.162 × 0.115 × 0.107
a (Å)	15.50430(10)	36.1492(6)
b (Å)	25.8431(2)	24.6871(4)
c (Å)	25.7164(2)	27.2753(4)
α (°)	90	90
β (°)	90.857(1)	101.4811(14)
γ (°)	90	90
V (Å^3)	10302.87(13)	23854.0(6)
Z	2	8
ρ_{calc} (g cm^{-3})	1.137	1.131
Radiation (Å)	CuKα (λ = 1.54184)	MoKα (λ = 0.71073)
μ (mm^{-1})	2.301	0.252
Reflections collected	79054	146759
Independent reflections	21299	27518
R_{int}	0.0369	0.0755
R_1 [$I > 2\sigma(I)$] [a]	0.0455	0.0546
wR_2 (all data) [b]	0.1302	0.1360
Goodness of fit on F^2	1.060	1.027
Largest diff. peak/hole (e Å^{-3})	1.354/–0.822	0.678/–0.460

(a) $R_1 = \Sigma||F_o|-|F_c|| / \Sigma|F_o|$, (b) $wR_2 = [\Sigma\{w(F_o^2-F_c^2)^2\} / \Sigma\{w(F_o^2)^2\}]^{0.5}$

Table S1. Crystallographic data (cont.)

	5
Formula	$C_{100}H_{160}Al_2N_2Na_2Nb_2O_{10}$, 4 (C_6H_6)
Formula Mass (g mol^{-1})	2148.48
Temperature (K)	123
Crystal system	*Monoclinic*
Space group	$P2_1/n$ (#14)
Crystal color	Red
Crystal size (mm)	0.208 × 0.132 × 0.129
a (Å)	13.5860(1)
b (Å)	20.7555(1)
c (Å)	21.8060(1)
α (°)	90
β (°)	94.998(1)
γ (°)	90
V (Å^3)	6125.57(6)
Z	2
ρ_{calc} (g cm^{-3})	1.165
Radiation (Å)	CuKα (λ = 1.54184)
μ (mm^{-1})	2.154
Reflections collected	45337
Independent reflections	12455
R_{int}	0.0433
R_1 [$I > 2\sigma(I)$] [a]	0.0315
wR_2 (all data) [b]	0.0828
Goodness of fit on F^2	1.051
Largest diff. peak/hole (e Å^{-3})	0.357/–0.696

(a) $R_1 = \Sigma||F_o|-|F_c|| / \Sigma|F_o|$, (b) $wR_2 = [\Sigma\{w(F_o^2-F_c^2)^2\} / \Sigma\{w(F_o^2)^2\}]^{0.5}$

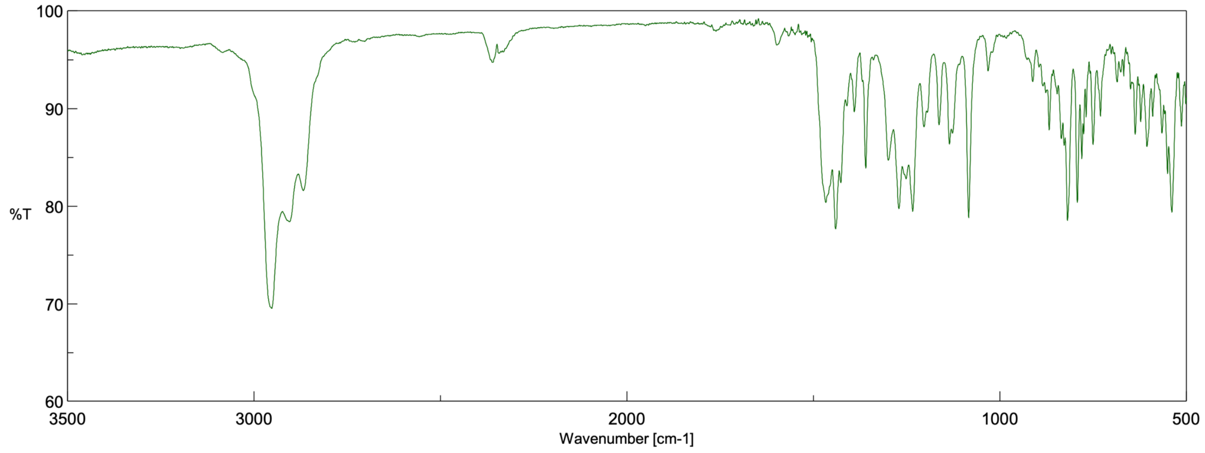

Figure S15. IR spectrum of **2-Na**.

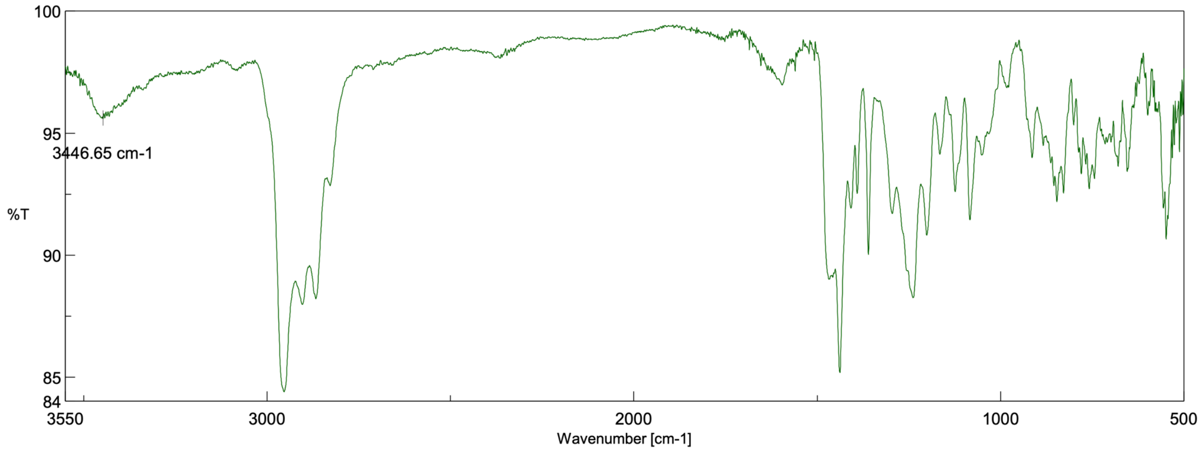

Figure S16. IR spectrum of **4**.

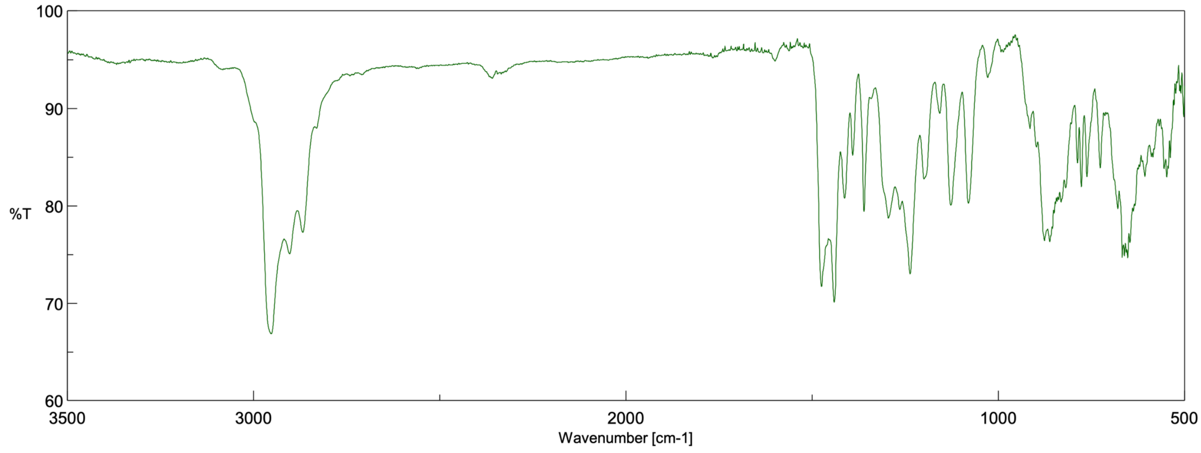

Figure S17. IR spectrum of **5**.
